# Items

**Published:** 1878-12

**Authors:** 


### Items.

—The recent trial, in Chicago, of Kennedy, the wife murderer, furnishes a novel medical development. The plea of insanity was entered by the defense. The medical experts (Drs. Lyman and Brower) swear they believed the man then insane, and that, in all likelihood, he was insane at the time of the homicide. It was in evidence that the defendant had frequently taken spirits of camphor in considerable quantity, to drive away, as he said, an evil spirit that dogged him.

The judge charged that if defendant was insane at the time of the homicide he was to be acquitted on that ground, *unless* the insanity was due to the use of alcohol, in which case the verdict must be of murder in the first degree. He did not say drunkenness, which seems not to have been proven, but insanity. The jury were also to state whether the evidence had established the present insanity of the prisoner.

The verdict was that of murder in the first degree, and that the punishment should be hanging; that the defendant, at the time of the trial, was insane.

It seems that the practice of all the courts has been, in such cases, to stop all proceedings against a prisoner who, at the date of his trial, is found insane.

Both the charge of the court and the verdict occasion snrprise.

As a sequel, the culprit, the next night after his trial, committed suicide in the jail by cutting his throat with a razor he had found in the corridor.

—*American female physicians*, whom we meet at every turn in Europe, have formerly been able to get private instruction in many special departments of medicine from the docents and assistants here. This winter, unfortunately for them and for those who may care after them, we have had several of the Mary Walker type, who were not contented with any privileges short of those accorded to men, and urged their claims with such pertinacity that the faculty were impelled to take action in the matter, which has resulted in excluding them entirely, in future, from everything except the lectures of Prof. Gustav Braun.

—A solid aqueous extract of ergot, prepared by Dr. E. R. Squibb, of Brooklyn, is available for hypodermic use, and is said not to be liable to cause local inflammation. Its strength is five times that of ergot, and it is administered in solution in water.

